# The complete mitochondrial genome of *Yaoshania pachychilus* (CHEN, 1980) (Cyprinifromes, Balitoridae)

**DOI:** 10.1080/23802359.2016.1155088

**Published:** 2016-03-28

**Authors:** Kang Xu, Fangzhou Hu

**Affiliations:** a Key Laboratory of Agro-ecological Processes in Subtropical Region, Hunan Provincial Engineering Research Center of Healthy Livestock, Institute of Subtropical Agriculture, Chinese Academy of Sciences, Changsha, People’s Republic of China;; b Key Laboratory of Protein Chemistry and Fish Developmental Biology of Education Ministry of China, College of Life Sciences, Hunan Normal University, Changsha, People’s Republic of China

**Keywords:** Cypriniformes, fish, mitochondrial genome, *Yaoshania pachychilus*

## Abstract

The complete mitochondrial genome of *Yaoshania pachychilus* (*Protomyzon pachychilus*) was determined for the first time. This mitogenome was 16 564 bp in length, including two ribosomal RNA genes, 22 transfer RNA genes, containing 13 protein-coding genes and a control region (D-loop). The base composition of the heavy strand was 28.9% A, 25.2% T, 28.7% C and 17.2% G. As in other vertebrates, with the exception of the eight tRNA genes and NADH dehydrogenase subunit 6 (ND6), all other mitochondrial genes are encoded on the heavy strand. Only the tRNA-Ser was not folded into a typical clover-leaf secondary structure as it lacks the dihydrouridine arm.

*Yaoshania pachychilus* (Teleostei, Cypriniformes, Homalopteridae, Protomyzon) is a rare fish found in China belonging to the family of Homalopteridae which has two subfamilies, a total of about 37 genera, 470 species (Yang et al. 2012). *Yaoshania pachychilus* is a demersal freshwater fish and it lives on hill rapids, the high mountain lakes and rivers. *Yaoshania pachychilus* is a small ornamental fish belonging to cold fish, and it has been listed in China red data book of endangered animals.

In this study, we have presented the complete mitochondrial genome of *Yaoshania pachychilus*. The specimens were collected from Dayaoshan Mountain, Jinxiu County, Xi Jiang, geographical coordinates: longitude 113°11′–113°16′, north latitude 25°07′–25°14′ of Guangxi Province, China. The primary specimens had been stored in herbarium in Kunming Institute of Zoology (KIZ), Chinese Academy of Sciences and the accession number is 200243055. This information can be queried in the National Specimen Information Infrastructure (NSII) of China. The complete mitogenome of *Yaoshania pachychilus* was 16 564 bp in size with 13 protein-coding genes, 22 tRNA genes and a control region (GenBank accession No. KT031050). Most of the mitochondrial genes were encoded on heavy strand except for NADH dehydrogenase subunit 6 (ND6) and eight tRNA genes (tRNA-Gln, -Ala, -Asn, -Cys, -Tyr, -Ser1, -Glu and -Pro) which were encoded on light strand (Boore 1999). The gene composition, arrangement and transcriptional orientation in *Yaoshania pachychilus* are similar to other vertebrates (Table 1). The overall base composition of the H-strand was 28.9% A, 25.2% T, 28.7% C and 17.2% G. All protein-coding genes started with the standard ATG codon except COX1 gene, which used GTG as the starting codon. TAA was the typical stop codon in seven protein-encoding genes, while TAG was the stop codon for protein-coding genes ND1, ND2, ND3 and ND4, and other two genes (COII and Cytb) had incomplete stop codon T. The 12S rRNA (952 bp) and 16S rRNA (1678 bp) genes were separated by the tRNA-Val gene. Twenty-two tRNA genes ranged from 66 bp (tRNA-Cys) to 76 bp (tRNA-Gly1). Only tRNA-Ser2 lacked the dihydrouridine arm and replaced it with a simple loop, the remaining tRNAs could be folded into a typical clover-leaf secondary structure (Liang et al. 2014). Between tRNA-Asn and tRNA-Cys, a 31-bp sequence was identified as the origin of L-strand replication (OL), which could be folded into a hairpin structure (8 bp stem and 13 bp loop). The A + T-rich (63.31%) control region (905 bp) was located between tRNA-Pro and tRNA-Phe. The termination-associated sequence (TAS) and the conserved sequence blocks (CSB1-3) were found in the control region.

**Table 1.** Characteristics of the mitochondrial genome of *Yaoshania pachychilus*

Gene	Start position	Stop position	Spacer (+), overlap (−)	Start code	Stop code	Size (bp)	Strand (sense)
*tRNA-Phe*(GAA)	1	69	0			69	H
*12sRNA*	70	1021	0			952	H
*tRNA-Val*(UAC)	1022	1094	0			73	H
*16sRNA*	1095	2772	0			1678	H
*tRNA-Leu1*(UAA)	2773	2847	+1			75	H
*ND1*	2849	3823	+5	ATG	TAG	975	H
*tRNA-Ile*(GAU)	3829	3900	−2			72	H
*tRNA-Gln*(UUG)	3899	3969	+1			71	L
*tRNA-Met*(CAU)	3971	4039	0			69	H
*ND2*	4040	5083	+1	ATG	TAG	1044	H
*tRNA-trp*(UCA)	5085	5155	+2			71	H
*tRNA-Ala*(UGC)	5158	5226	+1			69	L
*tRNA-Asn*(GUU)	5228	5300	0			73	L
*O*L	5301	5331	0			31	
*tRNA-Cys*(GCA)	5332	5397	+1			66	L
*tRNA-Tyr*(GUA)	5399	5466	+1			68	L
*C*ox1	5468	7018	+1	GTG	TAA	1551	H
*tRNA-Ser1*(UGA)	7020	7090	+2			71	L
*tRNA-Asp*(GUC)	7093	7165	+13			73	H
*C*ox2	7179	7868	+1	ATG	T	690	H
*tRNA-Gly1*(GCC)	7870	7945	+1			76	H
*A*TP8	7947	8114	−10	ATG	TAA	168	H
*A*TP6	8105	8788	−1	ATG	TAA	684	H
*Cox3*	8788	9573	−1	ATG	TAA	786	H
*tRNA-Gly2*(UCC)	9573	9644	0			72	H
*N*D3	9645	9995	−2	ATG	TAG	351	H
*tRNA-Arg*(UCG)	9994	10063	0			70	H
*N*D4L	10064	10360	−7	ATG	TAA	297	H
*N*D4	10354	11736	−1	ATG	TAG	1383	H
*tRNA-His*(GUG)	11736	11805	0			70	H
*tRNA-Ser2*(GCU)	11806	11873	+1			68	H
*tRNA-Leu2*(UAG)	11875	11947	0			73	H
*N*D5	11948	13786	−4	ATG	TAA	1839	H
ND6	13783	14304	0	ATG	TAA	522	L
*tRNA-Glu*(UUC)	14305	14373	+5			69	L
*C*ytb	14379	15515	+4	ATG	T	1137	H
*tRNA-Thr*(UGU)	15520	15591	−2			72	H
*tRNA-Pro*(UGG)	15590	15659	0			70	L
*D*-loop	15660	16564	0			905	

We had proceeded the molecular evolution analysis of mitochondrial DNA by using MEGA5.1 (Tamura et al. 2011). Phylogenetic analysis indicated that *Yaoshania pachychilus* had the closest relationship with *Metahomaloptera omeiensis* (Teleostei, Cypriniformes, Homalopteridae, Metahomalopterinae) (Li et al. 2014) which is a demersal freshwater fish (Figure 1).

**Figure. 1.**
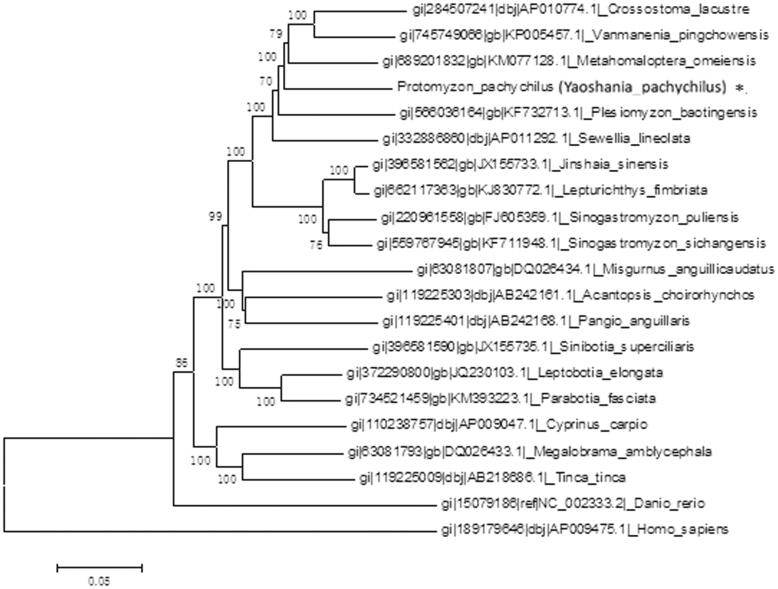
Phylogenetic tree was constructed using whole mitogenomes of *Yaoshania pachychilus* and other closely related organisms.

We think these data will contribute to uncover the related generic groups, the genetic and evolutionary relationships among Homalopteridae in the Cypriniformes in different areas of Chinese Mainland and Asia.

## References

[1] Yang J, Kottelat M, Yang JX, Chen XY. 2012 *Yaoshania* and *Erromyzon kalotaenia*, a new genus and a new species of balitorid loaches from Guangxi, China (Teleostei: Cypriniformes). Zootaxa. 3586:173–186.

[2] Tang WJ, Chen YY. 2000 Study on taxonomy of Homalopteridae. J Shanghai Fish Univ. 9:1–10 (in Chinese with English abstract).

[3] Li HJ, Zhao HX, Lin XT, Chen M. 2010 Fish communities in Dayaoshan State Natural Reserve of Guangxi, China. J Henan Normal Univ (Nat Sci). 38:123–127 (in Chinese with English abstract).

[4] Chen M, Huang N, Li HJ. 2002 Study on the fishes of Homalopteridae and their distribution from Guangxi. J Xinyang Teach Coll (Nat Sci Ed). 15:204–207 (in Chinese with English abstract).

[5] Boore JL. 1999 Animal mitochondrial genomes. Nucleic Acids Res. 27:1767–1780. 10101183 10.1093/nar/27.8.1767 PMC148383

[6] Liang Z, Wang C, Wu Y, Li H, Yuan X, Wei Q. 2014 Complete mitochondrial genome of *Vanmanenia pingchowensis* (Cypriniformes, Cyprinidae). Mitochondrial DNA. 27:1–2. 25427810 10.3109/19401736.2014.982617

[7] Tamura K, Peterson D, Peterson N, Stecher G, Nei M, Kumar S. 2011 MEGA5: Molecular Evolutionary Genetics Analysis Using Maximum Likelihood, Evolutionary Distance, and Maximum Parsimony Methods. Mol Biol Evol. 28:2731–2739. 21546353 10.1093/molbev/msr121 PMC3203626

[8] Li Y, Tang M, Xue Y, Chen HJ, Ye Q. 2014 Complete mitochondrial genome of the Chinese balitorine loach, *Metahomaloptera omeiensis* (Teleostei, Cypriniformes). Mitochondrial DNA. 27:1449–1450. 25162765 10.3109/19401736.2014.953087

